# Consortium profile: the methylation, imaging and NeuroDevelopment (MIND) consortium

**DOI:** 10.1038/s41380-025-03203-w

**Published:** 2025-09-06

**Authors:** Isabel K. Schuurmans, Rosa H. Mulder, Vilte Baltramonaityte, Alexandra Lahtinen, Fan Qiuyu, Leonardo Melo Rothmann, Marlene Staginnus, Jetro J. Tuulari, S. Alexandra Burt, Claudia Buss, Jeffrey M. Craig, Kirsten A. Donald, Johan G. Eriksson, Janine F. Felix, Tom P. Freeman, Rodrigo Grassi-Oliveira, Anke Huels, Luke W. Hyde, Scott A. Jones, Hasse Karlsson, Linnea Karlsson, Nastassja Koen, Will Lawn, Colter Mitchell, Christopher S. Monk, Michael A. Mooney, Ryan Muetzel, Joel T. Nigg, Síntia Iole Nogueira Belangero, Daniel Notterman, Yi Ying Ong, Tom O’Connor, Kieran J. O’Donnell, Pedro Mario Pan, Tiina Paunio, Peter Ryabinin, Richard Saffery, Giovanni A. Salum, Marc Seal, Tim J. Silk, Dan J. Stein, Ai Peng Tan, Ai Ling Teh, Dennis Wang, Heather Zar, Esther Walton, Charlotte A. M. Cecil

**Affiliations:** 1 Department of Child and Adolescent Psychiatry and Psychology, Erasmus MC University Medical Center Rotterdam, Rotterdam, the Netherlands; 2 Department of Psychology, University of Bath, Bath, UK; 3 Research Program in Systems Oncology, Research Programs Unit, Faculty of Medicine, University of Helsinki, Helsinki, Finland; 4 National Institute of Health and Welfare, Department of Public Health and Welfare, Population Health Unit, Helsinki, Finland; 5 Translational Neuropsychiatry Unit, Department of Clinical Medicine, Aarhus University, Aarhus, Denmark; 6 FinnBrain Birth Cohort Study, Turku Brain and Mind Center; Department of Clinical Medicine, University of Turku, Turku, Finland; 7 Centre for Population Health Research, Turku University Hospital and University of Turku, Turku, Finland; 8 Michigan State University, East Lansing, MI USA; 9 Development, Health and Disease Research Program, University of California, Irvine, CA USA; 10 Department of Pediatrics, University of California, Irvine, CA USA; 11 Department of Medical Psychology, Charité - Universitätsmedizin Berlin, Berlin, Germany; 12 Deakin University, IMPACT – The Institute for Mental and Physical Health and Clinical Translation, School of Medicine, Geelong, VIC Australia; 13 Murdoch Children’s Research Institute, Royal Children’s Hospital, Parkville, VIC Australia; 14 Department of Paediatrics, University of Melbourne, Royal Children’s Hospital, Parkville, VIC Australia; 15 Department of Paediatrics and Child Health, Red Cross War Memorial Children’s Hospital, University of Cape Town, Cape Town, South Africa; 16 Neuroscience Institute, University of Cape Town, Cape Town, South Africa; 17 Institute for Human Development and Potential (IHDP), Agency for Science Technology and Research (A*STAR), Singapore, Singapore; 18 Department of Obstetrics & Gynaecology, Yong Loo Lin School of Medicine, National University of Singapore, Singapore, Singapore; 19 The Generation R Study Group, Erasmus MC University Medical Center Rotterdam, Rotterdam, the Netherlands; 20 Department of Pediatrics, Erasmus MC, University Medical Center Rotterdam, Rotterdam, The Netherlands; 21 School of Medicine, Developmental Cognitive Neuroscience Lab, Pontifical Catholic University of Rio Grande Do Sul (PUCRS), Porto Alegre, Brazil; 22 Department of Epidemiology, Rollins School of Public Health, Emory University, Atlanta, GA USA; 23 Gangarosa Department of Environmental Health, Rollins School of Public Health, Emory University, Atlanta, GA USA; 24 Department of Biostatistics and Bioinformatics, Rollins School of Public Health, Emory University, Atlanta, GA USA; 25 Department of Psychology, University of Michigan, Ann Arbor, MI USA; 26 Center for Mental Health Innovation, Oregon Health & Science University, Portland, OR USA; 27 Department of Psychiatry, Oregon Health & Science University, Portland, OR USA; 28 Department of Psychiatry, Turku University Hospital & University of Turku, Turku, Finland; 29 Department of Clinical Medicine, Pediatrics and Adolescent Medicine, Turku University Hospital and University of Turku, Turku, Finland; 30 Department of Child Psychiatry, Turku University Hospital, Turku, Finland; 31 Department of Psychiatry and Mental Health, University of Cape Town, Cape Town, South Africa; 32 SAMRC Unit on Risk and Resilience in Mental Disorders, Cape Town, South Africa; 33 Department of Psychology, Institute of Psychiatry Psychology and Neuroscience, King’s College London, London, UK; 34 Institute for Social Research, University of Michigan, Ann Arbor, MI USA; 35 Knight Cancer Institute, Oregon Health & Science University, Portland, OR USA; 36 Federal University of São Paulo, São Paulo, Brazil; 37 Department of Molecular Biology, Princeton University, Princeton, NJ USA; 38 Department of Paediatrics, Yong Loo Lin School of Medicine, National University of Singapore, Singapore, Singapore; 39 University of Rochester Medical Center, Departments of Psychiatry, Psychology, Neuroscience, and Obstetrics and Gynecology, Rochester, NY USA; 40 Yale Child Study Center, Yale School of Medicine, New Haven, CT USA; 41 Department of Obstetrics, Gynecology, and Reproductive Sciences, Yale School of Medicine, New Haven, CT USA; 42 Sleepwell program and Department of Psychiatry, University of Helsinki and Helsinki University Hospital, Helsinki, Finland; 43 Department of Psychiatry, Universidade Federal do Rio Grande do Sul (UFRGS), Porto Alegre, Brazil; 44 Graduate Program in Psychiatry and Behavioral Sciences, Universidade Federal do Rio Grande do Sul (UFRGS), Porto Alegre, Brazil; 45 Child Mind Institute, New York, NY USA; 46 Developmental Imaging, Murdoch Children’s Research Institute, Parkville, VIC Australia; 47 School of Psychology and Centre for Social and Early Emotional Development (SEED), Deakin University, Geelong, VIC Australia; 48 Department of Diagnostic Radiology, Yong Loo Lin School of Medicine, National University of Singapore, Singapore, Singapore; 49 Bioinformatics Institute (BII), Agency for Science Technology and Research (A*STAR), Singapore, Singapore; 50 Department of Paediatrics & Child Health, Red Cross Children’s Hospital and SA-MRC Unit on Child & Adolescent Health, University of Cape Town, Cape Town, South Africa; 51 Molecular Epidemiology, Department of Biomedical Data Sciences, Leiden University Medical Center, Leiden, the Netherlands

**Keywords:** Neuroscience, Molecular biology, Psychology

## Abstract

Epigenetic processes, such as DNA methylation, show potential as biological markers and mechanisms underlying gene-environment interplay in the prediction of mental health and other brain-based phenotypes. However, little is known about how peripheral epigenetic patterns relate to individual differences in the brain itself. An increasingly popular approach to address this is by combining epigenetic and neuroimaging data; yet, research in this area is almost entirely comprised of cross-sectional studies in adults. To bridge this gap, we established the Methylation, Imaging and NeuroDevelopment (MIND) Consortium, which aims to bring a developmental focus to the emerging field of Neuroimaging Epigenetics by (i) promoting collaborative, adequately powered developmental research via multi-cohort analyses; (ii) increasing scientific rigor through the establishment of shared pipelines and open science practices; and (iii) advancing our understanding of DNA methylation-brain dynamics at different developmental periods (from birth to emerging adulthood), by leveraging data from prospective, longitudinal pediatric studies. MIND currently integrates 16 cohorts worldwide, comprising (repeated) measures of DNA methylation in peripheral tissues (blood, buccal cells, and saliva) and neuroimaging by magnetic resonance imaging across up to five time points over a period of up to 21 years (N_pooled DNAm_ = 12,877; N_pooled neuroimaging_ = 10,899; N_pooled combined_ = 6074). By triangulating associations across multiple developmental time points and study types, we hope to generate new insights into the dynamic relationships between peripheral DNA methylation and the brain, and how these ultimately relate to neurodevelopmental and psychiatric phenotypes.

## Background

DNA methylation (DNAm) is an epigenetic process that can regulate gene activity in response to both genetic and environmental influences [1, 2], beginning *in utero*. While DNAm plays a key role in *healthy* development and function [3], alterations in DNAm have also been linked to the emergence of *disease states*, including brain-based disorders such as neurodevelopmental, psychiatric, and neurological conditions [4–10]. In addition, peripheral markers of DNAm are often more accessible to measure than several brain-based phenotypes. Together, these properties make DNAm a promising molecular system in the search for both biological markers and mechanisms underlying gene-environment impacts on brain-based disorder and phenotypes. However, we still know little about how peripheral DNAm patterns relate to individual differences in the brain structure and function itself – the target organ of interest.

To address this gap, a growing number of researchers have examined associations between DNAm and brain features (typically measured using magnetic resonance imaging; MRI), giving rise to the new field of *Neuroimaging Epigenetics* [11–13]. Already more than 100 articles combining DNAm and brain MRI have been published to date, but important limitations remain. Most studies have assessed DNAm and the brain only at a single (cross-sectional) time point, are based on adult samples (~80%), have small to moderate sample sizes (median *N* = 98, range 14–715), and have primarily used a candidate gene approach (i.e., focusing on a limited set of CpGs; 67%). The first large-scale multi-cohort investigations using an epigenome-wide approach are now emerging; these generally show modest associations, with for example only two CpGs in blood found to be associated with hippocampal volume in a pooled analysis of 3337 participants after genome-wide correction [14].

## Why was the consortium set up?

Rapid advances in Neuroimaging Epigenetics have also been accompanied by several key challenges. First, studies in this field have been highly heterogeneous in terms of their design, characteristics, and methodology, with few shared practices, limiting comparability between findings and the identification of robust Neuroimaging Epigenetic associations. Second, the reliance on cross-sectional measurements, mainly in adults, likely ignores important temporal dynamics, as both DNAm and brain structure and volume are known vary over time [15–19] and in different ways (i) depending on the region examined (i.e., where in the genome or in the brain), (ii) in terms of their developmental trajectory (i.e., linear or non-linear), (iii) in light of (sensitive) periods with more change/growth (e.g., childhood and adolescence), (iv) considering the directionality of associations (i.e., whether changes in DNAm predict changes in the brain or vice versa), and (v) in association with different downstream phenotypes (e.g., neurodevelopmental, psychiatric). For example, recent evidence points to epigenetic *‘timing effects*’ [6–8], whereby DNAm patterns in cord blood at birth have been shown to prospectively associate with certain neurodevelopmental problems in childhood (e.g., ADHD symptoms [6, 7], social communication deficits [8]) *more strongly* than DNAm patterns measured cross-sectionally during childhood. Similar ‘timing effects’ have also been reported for changes in brain volume, with the strength of phenotypic associations varying depending on timing of assessment [20]. Yet, how DNAm and neuroimaging measures relate *to each other* at different developmental stages is largely unknown. Finally, studies to date are typically based on single cohorts with small sample sizes of *n* < 100, with limited statistical power to detect what are likely to be small associations. While neighboring fields have already showed how large-scale collaborative efforts between cohorts [21–24] can be achieved, such initiatives at the intersection of epigenetics, imaging and development are lacking.

Recently, we established The Methylation, Imaging and NeuroDevelopment (MIND) Consortium to address these gaps. Our overarching goal is to understand how peripheral DNAm patterns relate to variation in brain structure and function across development. Specifically, we aim to bring a developmental focus to the emerging field of Neuroimaging Epigenetics by (i) promoting collaborative, adequately powered developmental research via multi-cohort analyses; (ii) increasing scientific rigor through the establishment of shared pipelines and open science practices; and (iii) elucidating directionality of associations between DNAm and the brain via the use of prospective, longitudinal cohorts across development (from birth through emerging adulthood).

## Who is in the consortium and what has been measured?

**Fig. 1:**
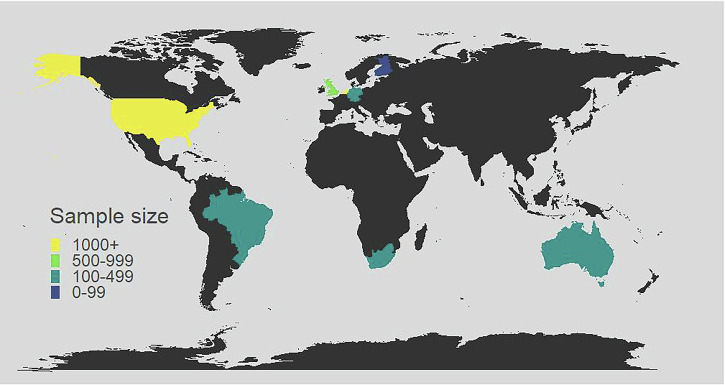
World map of sample size per country (overlap DNAm and neuroimaging), as covered in MIND. Sample sizes reflect *expected* sample size after sample processing (i.e., those featured in Table 1) and additionally include expected sample size for MTWinS (*N* = 708). Singapore is also included but too small to be shown on the map.

**Fig. 2:**
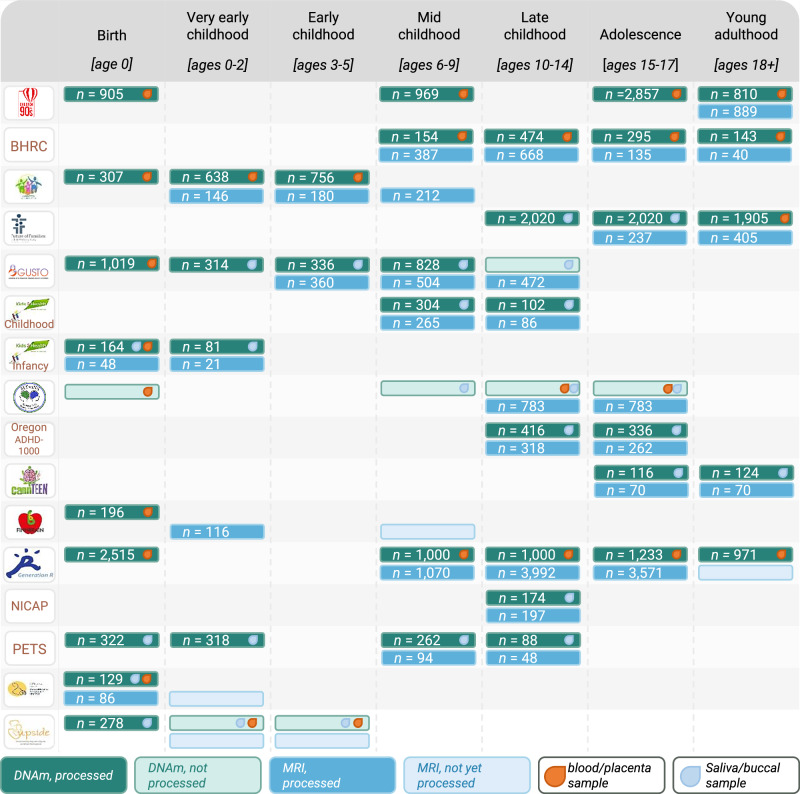
Time points and sample sizes of cohorts participating in MIND.

The MIND consortium brings together a global set of research teams and cohorts from North America, South America, Asia, Europe, African and Australia (Fig. 1). It currently includes 16 cohorts, spanning population-based, twin and high-risk cohorts. The unifying feature of these cohorts is the availability of genome-wide DNAm data (Illumina 450K or Illumina EPIC chip) and neuroimaging data (e.g., T1-weighted images, diffusion-weighted images, and/or resting state functional MRI) collected at one or more time points across development, with at least one assessment prior to age 18 years. Most cohorts have data from fetal life into childhood, with several cohorts also covering adolescence and early adulthood. With ongoing data collection in many cohorts, new DNAm and neuroimaging data are also expected to become available at additional time points. Key characteristics of cohorts included in the MIND consortium can be found in Table 1 and Fig. 2. Full cohort descriptions and study-specific acknowledgements can be found in the Supplementary Materials.

**Table 1:** Key characteristics of cohorts participating in MIND.

Cohort	Cohort abbreviation	PMID	Country	Study design	Total unique N		
					DNAm	Neuroimaging	Combined*
Avon Longitudinal Study of Parents and Children	ALSPAC	22507742	United Kingdom	Population-based birth cohort	3158	889	685
Brazilian High Risk Cohort	BHRC	25469819	Brazil	High-risk cohort	**	830	**
Drakenstein Child Health Study	DCHS	31751344	South-Africa	Population-based birth cohort	979	296	279
Future of Families and Child Wellbeing Study	FFCWS	35721627	United States	Population-based birth cohort	2020	483	483
Kids2Health – childhood	Kids2Health	-	Germany	High-risk cohort	417	420	407
Kids2Health – infancy	Kids2Health	-	Germany	High-risk cohort	102	86	85
Michigan Twin Neurogenetic Study	MTwiNS	31466551	United-States	Twin study, high-risk population-based birth cohort	**	708	**
Oregon ADHD-1000	Oregon ADHD-1000	32066674	United States	Community-recruited case-control cohort	752	580	496
The CannTeen Study	CannTeen	35772419	United Kingdom	High-risk cohort	240	140	133
The FinnBrain Birth Cohort Study	FinnBrain	29025073	Finland	Population-based birth cohort	196	116	97
The Generation R Study	GenR	28070760	The Netherlands	Population-based birth cohort	2532	5200	1893
The Neuroimaging of the Children’s Attention Project	NICAP	26969310	Australia	Community cohort, screening for ADHD	174	199	174
The Peri/Post-natal Epigenetic Twins Study	PETS	23171547	Australia	Twin study	322	100	100
UCIrvine Daily Experiences in Pregnancy Study	UCI cohort	28842114	United States	Community cohort	129	86	82
Understanding Pregnancy Signals and Infant Development	UPSIDE	33795306	United States	Community cohort	278	**	**

*Sample size combined indicates the unique sample size for each cohort, featuring participants with at least one DNAm measure, and one neuroimaging assessment. **Sample size not available, as data collection or processing is not finalized yet.

To date, the MIND cohorts comprise a total of 12,877 participants with DNAm profiles, 10,899 participants with neuroimaging data, and 6074 participants with both data types for neuroimaging epigenetic analyses. This is a significant expansion in sample size compared to the current average for *developmental* neuroimaging epigenetic studies (median *N* = 80, range 33–715 [11]), enabling us to characterize time-varying DNAm-brain associations in a more robust and nuanced manner. All cohorts have at least one time point of measurement, but 94% have two or more, up to five repeated time points. Cohorts participating in MIND are also notably diverse, including participants from various backgrounds in terms of ethnicity, geographical location and socio-economic environments, enabling the investigation of neuroimaging epigenetic associations across different settings, and increasing the inclusivity and generalizability of the findings. In addition, participants from most of the included cohorts have undergone extensive environmental, molecular and phenotypic profiling. As shown in Table 2, all cohorts have genetic data of participants – and in some cases also for relatives – which can be leveraged to account for population stratification, calculate polygenic scores, investigate gene-environment correlations and interactions as well as in some cases to study genetic nurture effects (i.e., using trio genetic data). Further, most cohorts have data on developmental and (mental) health outcomes, typically comprising measures of neurodevelopment, behavior, cognition and psychiatric symptoms, but also often feature other phenotypes such as anthropometrics and physical health assessments. Most cohorts have also measured environmental exposures, typically beginning in utero for the birth-cohorts (e.g., maternal smoking, diet, psychopathology and psychosocial stress during pregnancy) and during childhood/adolescence (e.g., early life stressors, parental influences, home environment and broader socio-economic factors). Finally, almost all cohorts have data on additional biological markers (e.g., inflammation) and profiling of other omics (e.g., microRNAs, metabolomics, proteomics and the gut microbiome).

**Table 2:** Overview of available measures different cohorts.

	Genetics	Other omics	Environmental and psychosocial exposures	Anthropometrics	Behavior and congnition
Avon Longitudinal Study of Parents and Children	Yes, in participants and relatives	Yes, inflammation markers, metabolomics and neuroendocrine markers	Yes, environmental and psychosocial exposures, and diet	Yes, height, weight, and body composition	Yes, behavior and cognition
Brazilian High Risk Cohort	Yes, in participants and relatives	Yes	Yes, psychosocial exposures	Yes, height and weight	Yes, behavior and cognition
Drakenstein Child Health Study	Yes, in participants	Yes, inflammation markers, microRNA, and gut microbiome	Yes, environmental and psychosocial exposures, and diet	Yes, height, weight, and body composition	Yes, behavior and cognition
Future of Families and Child Wellbeing Study	Yes, in participants and relatives	No	Yes, environmental and psychosocial exposures	Yes, height, weight, and body composition	Yes, behavior and cognition
Kids2Health – childhood	Yes, in participants	Yes, inflammation markers, metabolomics, gut microbiome, and neuroendocrine markers	Yes, environmental and psychosocial exposures	Yes, height, weight, and body composition	Yes, behavior and cognition
Kids2Health – infancy	Yes, in participants	Yes, inflammation markers, metabolomics, and gut microbiome	Yes, environmental and psychosocial exposures	Yes, height, weight, and body composition	Yes, behavior and cognition
Michigan Twin Neurogenetic Study	Yes, in participants	No	Yes psychosocial exposures	Yes, height and weight	Yes, behavior and cognition
Oregon ADHD-1000	Yes, in participants	No	Yes, environmental and psychosocial exposures	Yes, height and weight	Yes, behavior and cognition
The CannTeen Study	Yes, in participants	No	Yes, psychosocial exposures	Yes, height and weight	Yes, behavior and cognition
The FinnBrain Birth Cohort Study	Yes, in participants and relatives	Yes, gene expression, proteomics, metabolomics, inflammation markers, neuroendocrine markers, gut microbiome	Yes, psychosocial exposures	Yes, height and weight	Yes, behavior
The Generation R Study	Yes, in participants and relatives	Yes, inflammation markers, microRNAs, metabolomics, proteomics, gut microbiome, and neuroendocrine markers	Yes, environmental and psychosocial exposures, and diet	Yes, height, weight, and body composition	Yes, behavior and cognition
The Neuroimaging of the Children’s Attention Project	Yes, in participants	Yes, neuroendocrine markers	Yes, environmental and psychosocial exposures	Yes, height and weight	Yes, behavior and cognition
The Peri/Post-natal Epigenetic Twins Study	Yes, in participants	Yes, inflammation markers, gene expression, metabolomics, oral and gut microbiome.	Yes, environmental and psychosocial exposures, and diet	Yes, height, weight, and body composition	Yes, behavior and cognition
UCIrvine Daily Experiences in Pregnancy Study	Yes, in participants and mothers	No	Yes, environmental and psychosocial exposures, and diet	Yes, height, weight, and body compositions	Yes, behavior and cognition
Understanding Pregnancy Signals and Infant Development	Yes, in participants	Yes, inflammation markers, metabolomics, microbiome, and neuroendocrine markers	Yes, environmental and psychosocial exposures, and diet	Yes, height, weight, and body compositions	Yes, behavior and cognition

## How do we work together?

MIND operates on a federated model, wherein methods, practices, and results are shared among consortium members, while maintaining the confidentiality of the underlying individual-level data. Contributors have the flexibility to propose new projects, which operate on an opt-in basis. Participation from different cohorts in each project is optional and contingent on data availability, data sharing policies, resource allocation, and cohort-specific research interests. Similarly to consortia such as the Pregnancy And Childhood Epigenetics (PACE) Consortium [22], project leads are responsible for producing detailed analysis plans, which wherever possible will be pre-registered for transparency, and project leads will also be responsible for developing and testing analysis scripts. In the case of meta-analytic projects, scripts are disseminated across the consortium by the project lead for implementation by participating cohorts, who then share cohort-specific summary statistics with the project lead for pooling of results. Options for mega-analyses (i.e., pooled analyses of individual participant data) will also be explored in light of the increasing availability and uptake of federated data sharing platforms.

## Getting started: identifying and addressing challenges in collaborative research on developmental Neuroimaging Epigenetics

**Fig. 3:**
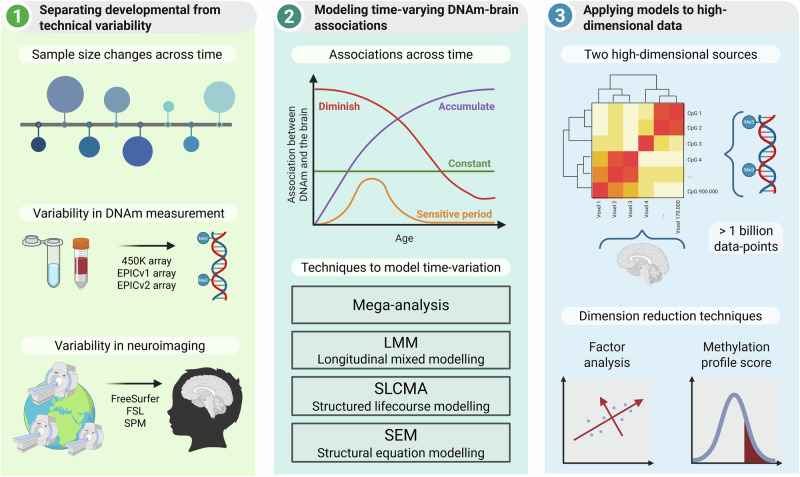
Key challenges in Neuroimaging Epigenetics. Created in https://BioRender.com.

Modeling (potentially) time-varying associations between two high-dimensional data modalities within a consortium framework offers new opportunities but also considerable challenges. Here, we outline three anticipated challenges within MIND, and reflect on potential strategies that could be used to address them (Fig. 3).

### Challenge #1: Separating developmental from technical variability

A key goal of MIND is to characterize neuroimaging epigenetic associations across development. We therefore plan to integrate data from cohorts covering different ages and developmental periods. While all cohorts in MIND have collected DNAm and neuroimaging data using comparable techniques, they still show substantial variability in sample characteristics and data processing methods. Notably, technical variability could increase generalizability of findings, e.g., MIND findings are more generalizable when using a range of methods, whereas studies using a single technique alone may be less generalizable to cases where different techniques are used. Yet, technical variability also complicates integration of different findings across cohorts. Furthermore, sources of technical variability across cohorts are tied to (or coincide with) differences in the timing of assessments, making it difficult to separate (developmental) signal from (technical) noise. This raises the challenge of balancing the need of maximizing statistical power (typically by increasing pooled sample size as much as possible) with maximizing comparability across diverse cohorts (instead calling for more focused pooling of data based on shared characteristics). We highlight below main sources of variability in sample characteristics, DNAm, and neuroimaging.

### Sample characteristics

MIND cohorts vary widely with respect to *sample size*, from numerous smaller studies with neonatal MRI (e.g., *N* = 86, UCI cohort) to larger cohorts with MRI data at different time points in childhood and adolescence (*N* = 822 at around age 18, ALSPAC cohort). This variation in sample size leads to uneven data availability across different developmental periods, resulting in unbalanced representation and differences in statistical power between these developmental periods. Furthermore, while almost all cohorts have repeated measures of DNAm or neuroimaging (or both), a small number has only a single time point available. This discrepancy results in *partial overlap* of samples when analyzing different developmental periods. In other words, the same group of participants is not consistently tracked across all time points in every study. To complicate matters, the ‘spacing’ of time points between DNAm and neuroimaging differs between cohorts. For example, while some cohorts have both DNAm and neuroimaging available in the neonatal period (maximum gap of a few weeks), others have DNAm at birth but only started with neuroimaging assessments in mid-childhood, resulting in a gap of several years. To address these sample differences, careful consideration of covariates (e.g., age at DNAm and neuroimaging assessments and the time gap between them) as well as methods to address partial sample overlap and sample size imbalance across cohorts (e.g., through the use of weighted approaches and leave-one-out analyses to test the stability of results) will be needed. Moreover, sex is a key covariate for consideration in DNAm-neuroimaging analyses. Studies have reported widespread autosomal sex differences in DNA methylation as early as at birth, with sex-differentiated sites enriched in genes related to nervous system development and mental health phenotypes [25, 26]. These differences may contribute to the observed sex disparities in psychiatric outcomes. Similarly, brain structure development also follows sex-dependent trajectories [27]. Given these findings, sex will be systematically accounted for in MIND analyses as a covariate, or where possible examined as a potential moderator through interaction analyses, to better understand the role of sex in DNAm-neuroimaging associations.

### DNAm

Another source of variability across studies is in how DNAm is measured, both in terms of DNAm array and tissue examined. The type of DNAm array used varies not only *between* MIND cohorts but at times also *within* longitudinal cohorts. The gold standard for DNAm assessment is whole-genome bisulfite sequencing (WGBS), offering single-nucleotide precision and covering about 95% of all CpGs on the genome, or roughly 28 million CpG sites [28]. However, its high cost and computational burden limits its use in large-scale cohorts. As an alternative, Illumina has developed a series of more affordable BeadChip microarrays, starting with the HumanMethylation27 BeadChip in 2008, expanding to over 450,000 CpGs with the 450 K array in 2011 [29], to over 850,000 CpGs with the EPICv1 in 2016 [30] and featuring an additional 186,000 CpGs with EPICv2 in 2023 [31]. The latest version, the Methylation Screening Array (MSA), contains over 284,000 probes of which only around 145,000 overlap with EPICv2 [32]. The cost-effective nature of these arrays (~3 times cheaper than WGBS) has made it possible for cohorts to measure hundreds of thousands of CpGs across the genome in larger samples, and as a result these methods have been eagerly adopted by many cohort studies. Yet, DNAm arrays and their technology change over time, and while these changes are marked by the removal of poor-quality sites [33] and addition of new probes with evidence for biological or clinical importance, the availability of data across several arrays presents challenges for longitudinal cohorts transitioning between versions. The discrepancies at the individual CpG level could reduce consistency in longitudinal research [34, 35]. Consequently, it may be difficult for longitudinal cohorts to distinguish between developmental changes *vs* variations due to array or batch-related factors, especially as measuring the same sample using multiple arrays is hardly ever done. Fortunately, studies comparing arrays have reported generally high concordance [31, 34–36].

Even within a single array, processing-related biases can arise, particularly due to batch effects linked to plate assignment. For instance, the 450 K BeadChip accommodates 12 samples per chip, with a standard processing plate holding 8 chips, totaling 96 samples being processed per plate. Meanwhile, the EPICv1 and EPICv2 arrays accommodate 8 samples per chip, with 8 chips per plate, allowing for a total of 64 samples being processed per plate. These processing plates can introduce systematic biases, which are especially problematic in longitudinal studies, where plate effects may become confounded with time points if all samples from a given time point are processed on the same plate. To mitigate this, studies planning to generate repeated measures of DNAm could apply a two-stage pseudo-randomization process, whereby (i) samples from the same individual at different time points are processed on the same plate, minimizing batch effects in within-subject longitudinal analyses, while also (ii) ensuring that individuals are adequately randomized across plates, to limit batch effects in between-subject analyses. If new time points are added to a cohort with an existing DNAm assessment, it would also be recommended to run ‘bridge samples’ (i.e. re-estimating a set of samples from the existing time point together with the new time points), in order to better quantify - and control for – resulting batch effects. This is for example the strategy that one of the MIND cohorts, Generation R, has recently applied to its new longitudinal epigenetic data (encompassing repeated DNAm measures at age 6, 10, 14, and 18 years), whereby repeated measures per individual were assigned to the same plate, and bridge samples were run to link this new resource to existing DNAm data at birth.

Another key challenge lies in the choice of tissue for DNAm measurement [37, 38]. Since in vivo DNAm assessment in brain tissue is not feasible, studies typically rely on peripheral DNAm (measured in more accessible tissues) to investigate associations with neuroimaging parameters. However, peripheral DNAm may not accurately capture brain methylation patterns, as only ~10% of CpG sites are estimated to be both variable in blood (range > 0.1) and concordant between blood and brain (based on correlation, r > 0.33, brain region-dependent) [38]. These CpGs can be identified using blood-brain methylation databases (e.g., BECon [38], Blood-Brain Comparison Tool [39], IMAGE-CpG [40]), which provide cross-tissue comparisons. Alternatively, significant findings can be validated using brain tissue derived from brain banks (e.g., The Netherlands brain bank for psychiatry [41]). However, in both cases, these data are primarily derived from adult postmortem brain samples and are typically limited to specific brain regions, leaving it unclear if these correlations generalize to the developmental context, in vivo, across different brain regions, or across different cell populations (neuronal versus non-neuronal). Efforts are emerging to profile DNAm in postmortem brains from fetal life onwards, although these studies have yet to establish the comparability of DNAm patterns between brain and more accessible peripheral tissues [42, 43]. Notably, even when cross-tissue correspondence is low, peripheral DNAm patterns may still be associated with brain features measured by MRI and hold biological relevance – for example, with blood-derived CpG sites reflecting inflammatory processes that influence brain morphology or connectivity. Additionally, such sites may have practical value in biomarker development, serving as indicators of risk factors for poor brain health, such as smoking.

In addition, there is also variability in across development [44]. At birth, DNAm is often measured from cord blood or other accessible tissues such as placenta, whereas later in life, samples are typically obtained from peripheral blood, saliva, or buccal cells. Even within the same tissue, cell-type variability may exist (e.g., cord blood versus peripheral blood). Most cohorts collect DNAm in only one tissue type, although MIND does feature a small subset of cohorts that collected DNAm in multiple tissues (e.g., saliva and blood, Kids2health). A common practice for many multi-cohort studies (e.g., [6, 14, 45]), has been to focus on blood tissue, including cord, heel prick and peripheral whole blood during development. While utilizing data obtained from a single tissue can enhance comparability and reduce noise between studies, it can come at the cost of lower sample size and limited generalizability to different tissues. Yet, even within the same tissue, cell-type composition can change dramatically across development, both in terms of the type and proportion of cells in bulk tissue. For example, nucleated red blood cells (nRBCs) are abundant in cord blood, but largely absent in blood later in development. Cell-type composition in bulk tissue can be adjusted for using age/tissue-appropriate reference panels (e.g., reference for cord blood versus peripheral blood at later time points, [6, 45]). However, this also introduces a further source of technical variability in longitudinal analyses (given that each panel has been estimated using different data sources). For example, a previous study characterizing DNAm trajectories across development found widespread age-related changes in DNAm patterns over the first two decades of life [15]. In some cases, these changes were non-linear (e.g. DNAm changing more rapidly from birth to mid-childhood, compared to later time points), raising questions about whether these changes are due to developmental timing or differences between cord and peripheral blood. In this respect, the creation of a single, integrated panel which estimates a more comprehensive set of cell-types, including (i) cells that may be present at only one time point (e.g., nRBCs at birth), as well as (ii) cell types that may be ubiquitous across development, but still vary in abundance over time (e.g., naïve versus memory B cells), could offer an attractive solution to better separate tissue from timing-related differences.

### Neuroimaging

Measurement heterogeneity is also a factor for neuroimaging assessments. MRI scanner types typically vary across study sites, and even within *one* site, scanners are often upgraded or replaced over time. Different strategies that have been proposed for reducing this unwanted geographical and temporal variation, include harmonization techniques like ComBat [46–48], deep learning [49], or hierarchical Bayesian regression [50]. However, removing the geographical and temporal variation from neuroimaging assessments can be challenging in developmental research: consider participants scanned repeatedly at different ages (e.g., as newborns and later in development) at various locations or with different scanners. While harmonization techniques aim to eliminate unwanted scanner or site variations, there is a risk they might also inadvertently remove developmental variation-of-interest. Currently, there is a lack of consensus about whether these methods should or should not be used for developmental cohorts. For example, one large-scale study by Ge et al. [19], involving almost 40,000 participants from 86 cohorts, used ComBat to control for unwanted site effects while comparing statistical methods for normative modeling of brain morphometric data. Another large-scale study by Kia et al. [50], also including almost 40,000 participants from 79 scanners, harmonized their data by using federated hierarchical Bayesian regression. In contrast, another large study by Bethlehem et al. [18], including over 100,000 participants from 100 cohorts, chose not to perform harmonization techniques for constructing normalized brain charts. Overall, it is unclear how best to strike a balance between reducing unwanted scanner variation while also preserving developmental changes crucial for accurate interpretation.

Most neuroimaging processing software packages - such as FreeSurfer, Statistical Parametric Mapping (SPM), and FMRIB Software Library (FSL) – have been developed for and tested in *adult* samples, raising concerns about how these methods perform in data from *infants and children*. For example, although the standard FreeSurfer suite has been successfully applied to data from children aged 4 years [51], *Infant FreeSurfer* has been developed for infants aged 0–2 [52]. Other software packages developed especially for fetal or neonatal brain scans include iBEAT [53] and NEOCIVET [54]. An important question that follows is whether cohorts should prioritize methodological consistency (i.e., reduce technical variability at the cost of potentially lower accuracy, by processing all neuroimaging data with the same software) or developmental sensitivity (i.e., use accurate tools at the cost of methodological consistency, by processing neuroimaging data with developmentally appropriate software) when harmonizing cross-cohort neuroimaging data across different life stages. Both strategies have been used in the past. For example, a recent analysis from the ENIGMA Lifespan consortium, including data from age 3 to 90 years across 86 cohorts, only used standard FreeSurfer [19]. In contrast, Bethlehem et al. [18] created normalized brain charts for mid-gestation up to age 100, while allowing for the use of developmentally-sensitive processing software across cohorts. Consequently, all adult cohorts were processed using FreeSurfer, but there was considerable heterogeneity of methods in data from pre-birth to early childhood (e.g., manual segmentation, NEOCIVET, customized FreeSurfer versions, Infant FreeSurfer, and standard Freesurfer). It is still unclear whether the use of age-tailored software tools adds or reduces noise in longitudinal analyses that span several developmental periods (e.g., birth to early adulthood). Other strategies include leveraging age-appropriate software, while clustering analyses by developmental period, and accounting for software-related variations during the meta-analysis phase. Alternatively, findings can be triangulated using different processing software. Overall, research evaluating the best options for leveraging developmentally sensitive data is needed to provide evidence-based guidelines for the field.

### Challenge #2: Modeling time-varying DNAm-brain associations in multi-cohort analyses

Besides considering how best to account for variability between MIND cohorts, decisions must also be made regarding how best to statistically perform multi-cohort integration. Both DNAm and brain structure are highly dynamic across development, meaning their associations may differ depending on when they are assessed [15–19]. In consortium settings, standard meta-analyses remain the most popular option, where an exposure (e.g., DNAm at specific loci) is associated with an outcome (e.g., a brain feature) using regression models within each individual cohort, after which cohort-level summary statistics are pooled through meta-analysis. Such methods were used by Jia et al. [14], which is currently the largest DNAm-brain multi-cohort study [12], meta-analyzing data from 3337 participants (mainly adults) across 11 cohorts. Notably, only two CpGs were found to be associated with hippocampal volume after correction for multiple testing, and none for the thalamus or the nucleus accumbens – the other two regions investigated. While this could suggest that the meta-analysis was either underpowered to detect modest effects or that peripheral DNAm and volumetric brain differences are largely unrelated, it could also indicate that rather than being constant, associations vary over time. Unless explicitly modeled, however, this information is lost in standard meta-analytic approaches, which inherently assume constant exposure-outcome associations; i.e., that the link between DNAm and the brain does not change over time. Although this restriction could have the advantage of identifying age-averaged associations (which may be more generalizable across age groups), it comes at the cost of obscuring time-varying effects. This may be particularly problematic for studies which pool data from both pediatric and adult cohorts, as changes in DNAm patterns and brain features are expected to be more drastic earlier in life.

An alternative approach to address developmental timing in multi-cohort studies involves strategic pooling of summary data at specific age ranges, as opposed to pooling all cohorts together regardless of age. This approach is commonly used in the PACE consortium (e.g., [6, 45]), prioritizing one epigenetic time point of interest (e.g., birth, because it is generally the time point with the largest sample size, or because of a focus on pregnancy exposures) and then quering later DNAm assessments by (i) adopting a follow-forward approach, where significant CpG sites are selected for further analysis and tested at subsequent time points (e.g., [7, 9, 55]) or by (ii) conducting a completely new set of analyses at later developmental stages (e.g., running separate epigenome-wide analyses using DNAm at birth and DNAm in childhood in relation to the same child outcome [6, 45]). While this strategy helps to partition analyses according to more developmentally-specific age groups, it does not directly quantify temporal changes in DNAm-brain associations, nor does it account for the potential non-linearity of this relationship over time. For this, meta-regression methods could be applied, which allow to test, within the same model (still based on cohort-specific summary statistics), (i) how DNAm and brain features associate at each available time points/developmental period, (ii) whether these associations change across time periods, and (iii) whether changes are linear or non-linear [15–17]. For an example of applying longitudinal meta-regression to quantify DNAm timing effects on child health outcomes using cohort-level summary statistics from the PACE Consortium, see [56].

While the above strategies can help us get started, ultimately, the transition toward a *mega*-analysis framework using individual-level data will offer greater modeling flexibility and new opportunities to address developmental questions in collaborative multi-cohort studies. Three concrete examples of potential applications here include longitudinal mixed models (LMM), structured life-course modeling analysis (SLCMA) and structural equation modeling (SEM). Mixed models test whether *changes* in a predictor (measured at repeated time points, e.g., DNAm at birth, age 6, and age 10) associates with an outcome (measured at single time points, e.g., brain feature at age 14) over time. While we are not aware of any existing *multi*-cohort studies using LMM to model DNAm-brain associations, this method was recently applied (i) in a single-cohort study to examine whether DNAm sites across time prospectively associate with the amygdala:hippocampus ratio in early adulthood [57], and (ii) to individual-level data from two longitudinal cohorts in order to characterize how epigenome-wide DNAm patterns vary over the first two decades of life [15]. As an alternative to LMM, SLCMA uses repeated measures of a *predictor* to test competing temporal hypotheses about its effect on a particular outcome (measured at a single time point). It has been used for example to test the developmental effect of stress exposure on child DNAm, showing that stressful experiences are more likely to associate with changes in DNAm if they occur early rather than late in childhood [58, 59]). This approach could be extended to model repeated DNAm as a predictor, in order to establish whether epigenetic effects on brain outcomes may also be developmentally-specific (orange line, Fig. 3), or might diminish or accumulate over time (red and purple lines, Fig. 3), as opposed to being temporally invariant (green line, Fig. 3). Finally, SEM allows modeling of repeated measures for *both* the predictor and the outcome; it has been previously applied to DNAm and neuroimaging data, although not within the same study. In the context of MIND, this model could be used to integrate DNAm-brain data, for example, (i) to estimate the degree of temporal stability in either epigenetic patterns and brain features across development (i.e., autoregressive paths), (ii) to examine relationships between these DNAm and the brain over time (i.e., cross-lagged paths), and (iii) to test genetic/environmental predictors or phenotypic (health) outcomes of identified DNAm-brain associations, as well as mediating effects (i.e., indirect paths). Despite the potential of these advanced methods to model complex (repeated) DNAm-brain measures for time-varying associations, their implementation within a multi-cohort framework remains a more distant goal. Success in this respect will first depend on overcoming existing barriers in sharing of individual-level data, increasing availability of meta-analyses for advanced statistical models, accessing a large enough set of cohorts with sufficiently comparable repeated DNAm/neuroimaging measures, and deciding how best to balance the complexity of longitudinal methods with the high-dimensionality of these data types, as discussed in more detail below.

### Challenge #3: Analyzing high-dimensional data

A third set of challenges relates to the dimensionality of DNAm and MRI data. In MIND, if we were to focus on single predictor–single outcome models, with a sample size of approximately >6000, we would be powered to detect an effect size (R^2^) of 0.001 at *p* < 0.05. However, DNAm data is commonly investigated through univariate epigenome-wide associations (with covariate adjustment), where DNAm-phenotype relationships are studied for a large number of individual sites across the genome. Epigenome-wide investigations currently interrogate up to 900,000 data points per person, depending on which array is used (note that even more data points can be measured with some technologies [28]). A commonly employed statistical threshold in such studies is *p* < 9 × 10⁻^8^ [60]. Under this threshold, MIND would be powered to detect associations with an effect size (R^2^) of 0.006. Yet, brain data can be treated as high-dimensional as well, and are often examined voxel-wide, where local density of gray matter is compared between participants (e.g., per 1mm^3^ of the brain), again resulting in hundreds of thousands of data points per participant. Findings from other epigenetic or neuroimaging consortia already suggest that detecting small to medium effects typically requires thousands of participants to ensure robust and reproducible associations when analyzing epigenetic these data types independently [61, 62]. When these datasets are combined, the challenge of collating adequately powered sample sizes becomes even greater. If epigenome- and voxel-wide data are analyzed together in the same model, even if just at a single time point, this would add up to examining more than a billion associations (CpG by voxel). At present, there are no concrete estimates for the sample sizes required for such large-scale Neuroimaging Epigenetics analyses. However, assuming a strict Bonferroni-adjusted threshold of *p* < 1 × 10⁻^13^ (as based on genome by voxel-wide analyses [63]), we would still be powered to detect small effects with an R^2^ of 0.011 with an sample size >6000. Notably, recent efforts have been made to apply such high-dimensional models within a single time point in the context of gene-environment interplay on DNAm, with some promising findings [64]. Yet, such models, especially if extended longitudinally, are currently neither practical (due to computational burden) nor feasible (due to limited power in smaller subsets of the data) within Neuroimaging Epigenetics. Therefore, in the context of MIND, some level of data manipulation is necessary.

One approach to managing high-dimensional DNAm data is preselecting CpGs, e.g. by focusing on variable CpGs known to differ across individuals, those with known cross-tissue correspondence (identified within blood-brain methylation databases [38]), or by focusing on CpGs close to genes implicated in neuronal development. However, candidate-based approaches have inherent limitations, including reduced discovery potential, the risk of overlooking novel or developmentally relevant CpG sites, and the challenge of ensuring generalizability across diverse populations and tissues. Alternatively, high-dimensional data can be handled by applying dimension reduction techniques, such as principal component analysis, (parallel) independent component analysis, local Fisher’s discriminant analysis, and canonical correlation analysis [65, 66]. These methods have indeed been applied to neuroimaging epigenetic data in single cohorts, although their application to multi-cohort analyses can be challenging due to the potential of obtaining different model solutions (e.g., factors or components comprised of different CpGs/brain features, or different weights) in different cohorts.

Additionally, we can condense high-dimensional data into singular aggregate scores that can be computed in a comparable way across cohorts, as done for example in the case of biological age estimates such as epigenetic age and brain age [67, 68]. In particular, the use of methylation profile scores (MPS) is gaining popularity in epigenetic research [69], following in the footsteps of polygenic scores (PGS) within the field of genetics. Within MIND, we could use MPSs to proxy at an epigenetic level brain-relevant exposures (e.g., prenatal smoking or insufficient sleep), biological processes (e.g., inflammation, neuroendocrine function), and health outcomes (e.g., mental or cardiometabolic phenotypes). We could also seek to develop MPSs of brain features themselves, an approach not yet attempted. In this context, it would be particularly interesting to conduct pathway analyses on the included CpGs to identify the underlying biological mechanisms driving associations in Neuroimaging Epigenetics. Another application of MPSs is to use them as a proxy for missing covariates or to enhance the quality of data imputation, which could be particularly helpful in the case of multi-cohort analyses (as certain cohorts may not have collected important covariates, or at least not at the specific age of interest). Overall, MPSs may offer a practical solution to manage high dimensionality while also aggregating together many epigenetic loci of small effect; yet, they also have their challenges. For example, MPSs are increasingly constructed using penalized linear regression techniques, which require adequate consideration and availability of training, tuning and testing datasets. The selection and splitting of datasets, and the amount of data reserved for training versus testing, can reduce sample size and power. Of note, to obtain reliable estimates for PGSs in complex traits, discovery samples from tens to hundreds of thousands of individuals have been required. Sample sizes required to construct reliable MPSs remain undefined. Furthermore, the predictive power of PRSs is influenced by ancestry [70]. While some evidence shows this may be less of an issue with MPSs [71], it will be of interest to test performance of MPSs in MIND to generate MPSs that can ideally be applied across ancestries. Finally, MPSs cannot be reliably constructed without accounting for key covariates known to influence the epigenome, such as age, sex, prenatal and postnatal exposure to smoking, and cell-type composition. All these factors must be carefully adjusted for or integrated into model construction to ensure valid MPSs.

## Conclusions

The potential of large-scale collaborative efforts between cohorts has been clearly demonstrated by the success of genome-wide association studies (for example by the Psychiatric Genetic Consortium [21]), epigenome-wide association studies (for example by PACE [22]), and neuroimaging studies (for example by ENIGMA [23] or specifically ENIGMA-ORIGINs [24]). The MIND consortium aims to apply these principles to the emerging field of Neuroimaging Epigenetics, with a particular emphasis on development. Priorities include fostering multi-cohort analyses to access collaborative, adequately powered developmental research; to establish shared pipelines; and to elucidate the time-varying relationship between peripheral DNAm and brain development through prospective, longitudinal cohorts spanning pre-birth to young adulthood. MIND is committed to pursuing these goals while supporting open science practices, including the use of pre-registration of studies, sharing of analysis scripts, and making full results (e.g., meta-analysis summary statistics) openly accessible. Through these activities, the consortium aims to facilitate an integrative and efficient research environment and to enhance the transparency and reproducibility of our research. Overall, we hope that MIND will represent a significant step towards shedding light into the complex, dynamic relationship between peripheral DNAm and the brain, and to understanding how these ultimately relate to neurodevelopmental and psychiatric phenotypes.

## Joining us

The MIND Consortium operates as an open network, welcoming researchers interested in joining with one or more of its cohorts. In this consortium, each participant manages and analyses their data locally. Detailed protocols for each study can be accessed through their individual websites (see Supplementary materials) or by contacting the study investigators. In order to participate in MIND, cohorts must feature at least one assessment of DNAm (peripheral, array-based) and neuroimaging collected before the age of 18 years. Those interested in collaborating with the MIND Consortium are encouraged to contact the corresponding authors (Dr. Esther Walton, E.Walton@bath.ac.uk; Dr. Charlotte Cecil, c.cecil@erasmusmc.nl). More information can also be found on our website: https://www.erasmusmc.nl/en/research/groups/methylation-imaging-and-neurodevelopment-mind-consortium#.

## Supplementary information

Supplementary materials
